# Development of a novel, robust and cost-efficient process for valorizing dairy waste exemplified by ethanol production

**DOI:** 10.1186/s12934-019-1091-3

**Published:** 2019-03-11

**Authors:** Jing Shen, Jun Chen, Peter Ruhdal Jensen, Christian Solem

**Affiliations:** National Food Institute, Technical University of Denmark, 2800 Kongens Lyngby, Denmark

**Keywords:** *Corynebacterium glutamicum*, Lactose-utilization, Medium optimization, Ethanol production

## Abstract

**Background:**

Delactosed whey permeate (DWP) is a side stream of whey processing, which often is discarded as waste, despite of its high residual content of lactose, typically 10–20%. Microbial fermentation is one of the most promising approaches for valorizing nutrient rich industrial waste streams, including those generated by the dairies. Here we present a novel microbial platform specifically designed to generate useful compounds from dairy waste. As a starting point we use *Corynebacterium glutamicum*, an important workhorse used for production of amino acids and other important compounds, which we have rewired and complemented with genes needed for lactose utilization. To demonstrate the potential of this novel platform we produce ethanol from lactose in DWP.

**Results:**

First, we introduced the *lacSZ* operon from *Streptococcus thermophilus*, encoding a lactose transporter and a β-galactosidase, and achieved slow growth on lactose. The strain could metabolize the glucose moiety of lactose, and galactose accumulated in the medium. After complementing with the Leloir pathway (*galMKTE*) from *Lactococcus lactis*, co-metabolization of galactose and glucose was accomplished. To further improve the growth and increase the sugar utilization rate, the strain underwent adaptive evolution in lactose minimal medium for 100 generations. The outcome was strain JS95 that grew fast in lactose mineral medium. Nevertheless, JS95 still grew poorly in DWP. The growth and final biomass accumulation were greatly stimulated after supplementation with NH_4_^+^, Mn^2+^, Fe^2+^ and trace minerals. In only 24 h of cultivation, a high cell density (OD_600_ of 56.8 ± 1.3) was attained. To demonstrate the usefulness of the platform, we introduced a plasmid expressing pyruvate decarboxylase and alcohol dehydrogenase, and managed to channel the metabolic flux towards ethanol. Under oxygen-deprived conditions, non-growing suspended cells could convert 100 g/L lactose into 46.1 ± 1.4 g/L ethanol in DWP, a yield of 88% of the theoretical. The resting cells could be re-used at least three times, and the ethanol productivities obtained were 0.96 g/L/h, 2.2 g/L/h, and 1.6 g/L/h, respectively.

**Conclusions:**

An efficient process for producing ethanol from DWP, based on *C. glutamicum*, was demonstrated. The results obtained clearly show a great potential for this newly developed platform for producing value-added chemicals from dairy waste.

**Electronic supplementary material:**

The online version of this article (10.1186/s12934-019-1091-3) contains supplementary material, which is available to authorized users.

## Background

Bioethanol is currently one of the most important biofuels [1], the worldwide production of which reached 100 billion liters in 2016 (Renewable Fuels Association, 2016). Sugars derived from corn and sugar cane are commonly used as feedstocks to produce bioethanol [2], and thus bioethanol production is directly competing with food production for land use [3]. Second-generation approaches for producing bioethanol rely on different alternative feedstocks, e.g., lignocellulosic biomass or woody crops, agricultural residues or waste [4], and could be a better alternative. There has been a lot of research focusing on a variety of subjects in the area, e.g. on biomass treatment, strain development, and process optimization, to facilitate efficient production of second-generation bioethanol from different waste streams [5–8].

Cheese whey, a byproduct of the dairy industry, represents a major environmental problem due to the large amounts produced and its rich nutritional composition resulting in a high biochemical oxygen demand (BOD) and a high chemical oxygen demand (COD). The annual worldwide whey production is estimated at around 190 million ton [9]. Whey basically contains 4–5% lactose, 1% protein, 0.4% lipids and small amounts of minerals and vitamins [10]. Most of the protein and lactose in whey can be separated by membrane filtration, and the concentrates of proteins and lactose are used as functional food ingredients. DWP, a concentrated residual of the process, contains 10–20% lactose and minerals, is of little economic value but of high polluting load, and is commonly used as animal feed [11]. As DWP still contains large amounts of lactose, efforts have been put to develop valorization processes, for instance, processes where the sugar is converted into valuable chemicals through microbial fermentation [12–15].

Converting lactose into ethanol is not novel, and can be done using non-conventional yeasts such as *Kluyveromyces lactis*, *Kluyveromyces marxianus* and *Candida pseudotropicalis*, which have the ability to metabolize lactose [14, 16, 17]. *Saccharomyces cerevisiae* (*S. cerevisiae*), the workhorse of industrial ethanol production, lacks a functional lactose metabolism [18], but can be used if lactose first is hydrolyzed to glucose and galactose [19], however, the concurrent release of glucose and galactose causes catabolite repression, which prolongs the fermentation process [20]. Recombinant *S. cerevisiae* strains that can utilize lactose have been generated [21], but most strains displayed undesirable characteristics, such as slow growth, genetic instability or problems derived from the use of glucose and galactose mixtures [22, 23]. *Escherichia coli* (*E. coli*) is an important model organism, and it is also capable of metabolizing lactose naturally, so many efforts have been carried out to build recombinant *E. coli* for ethanol production from lactose [24, 25]. Recently we demonstrated efficient ethanol production from whey waste using an engineered *Lactococcus lactis* (*L. lactis*) strain. *L. lactis* [26], like most other lactic acid bacteria, is fastidious in nature, and requires different nutrients in order to grow well, which potentially could be a drawback for industrial production.

*Corynebacterium glutamicum* is a rapidly growing, generally recognized as safe (GRAS) Gram-positive bacterium. It has been the workhorse for industrial production of amino acids and nucleotides for several decades [27]. More recently it also has been metabolically engineered into a robust and efficient cell factory for the production of bulk chemicals such as succinic acid, isobutanol, and ethanol [28]. For ethanol, this was accomplished by expressing the pyruvate decarboxylase gene (*pdc*) and the alcohol dehydrogenase gene (*adhB*) from *Zymomonas mobilis* (*Z. mobilis*) in different strain backgrounds, and more than 10% (vol.%) ethanol could be made at a high yield (94%) using a mineral salt medium [29, 30]. However, *C. glutamicum* lacks a β-galactosidase and is unable to metabolize lactose [31], and thus is not immediately a good candidate as a platform for converting lactose-containing waste into value-added chemicals. When the *lacY* and *lacZ* genes from *E. coli* were expressed in *C. glutamicum* R163, growth on the glucose moiety of lactose was possible, but galactose remained and accumulated in the medium [31]. Barrett et al. heterologously expressed both the lactose- and galactose-utilizing pathways from lactic acid bacteria in *C. glutamicum* and successfully employed the engineered *C. glutamicum* to produce l-lysine on a whey-based medium. However, this strain exhibited slow growth on lactose, and the plasmid-based expression vector was unstable [32]. These results indicate that further work is needed before a robust *C. glutamicum* strain capable of efficiently transforming dairy waste into valuable products is ready.

**Fig. 1:**
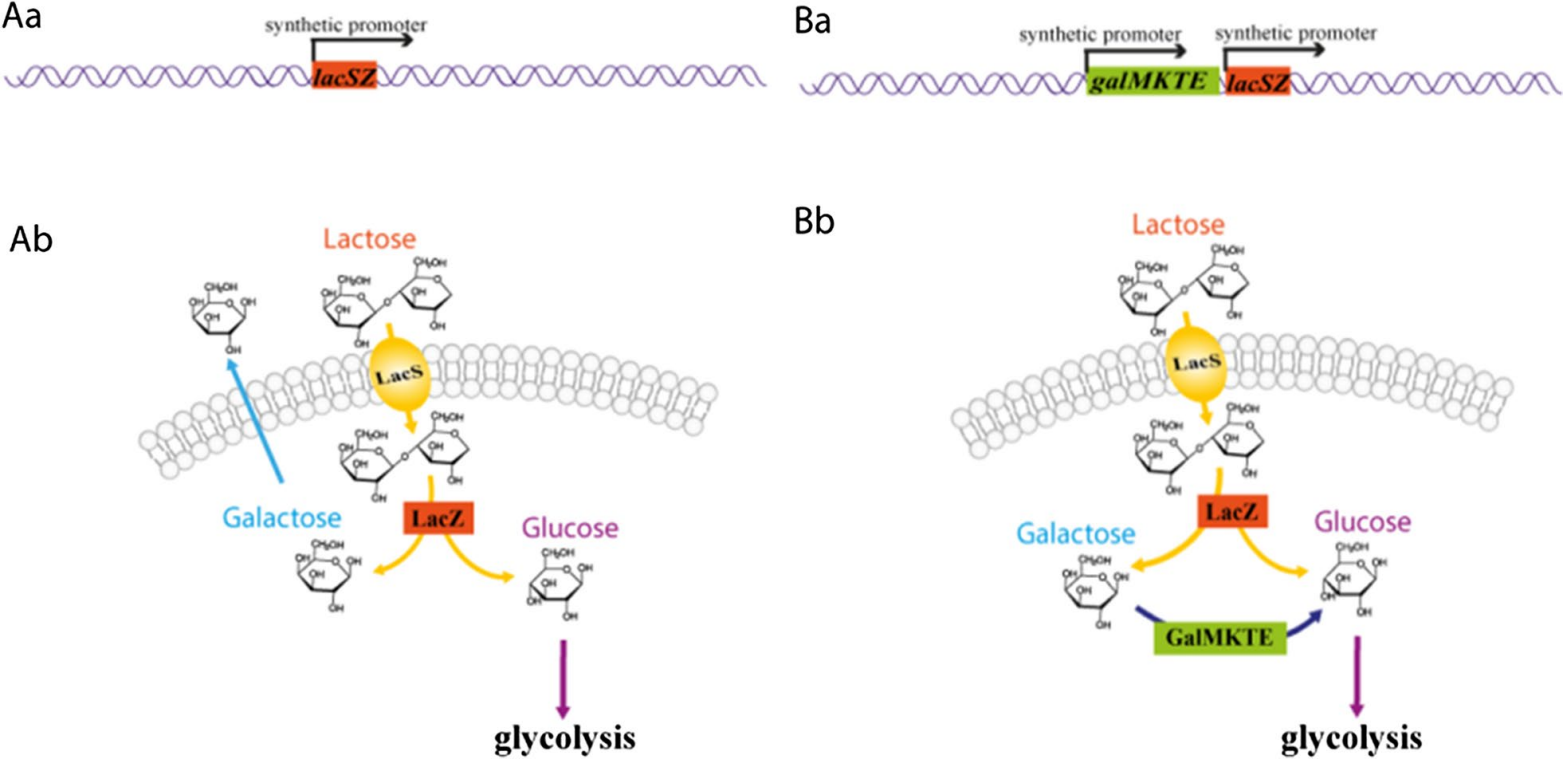
Construction of the lactose-metabolizing *C. glutamicum* strain. **Aa** Introduction of the *lacSZ* operon into the chromosome of *C. glutamicum*; **Ab** lactose catabolism in the *C. glutamicum* strain with integrated *lacSZ* operon; **Ba** additional chromosomal introduction of the *galMKTE* operon; **Bb** lactose catabolism in the *C. glutamicum* strain harboring both the *lacSZ* and *galMKTE* operons

In this study, we have constructed a derivative of *C. glutamicum* which is capable of metabolizing lactose. We have done this by introducing genetic elements from two lactic acid bacteria, namely *S. thermophilus* and *L. lactis*, by using a recently developed chromosomal integration tool [33] (see Fig. 1A, B). Subsequently, we rely on adaptive laboratory evolution, to speed up growth and lactose metabolization rate, and obtain an efficient platform for valorizing dairy waste. We demonstrate the latter by producing ethanol from the lactose in DWP.

## Results

### Construction of a lactose metabolizing *C. glutamicum* strain and assessment of its genetic stability

To enable *C. glutamicum* to grow on lactose, we decided to introduce the *lacSZ* operon from *S. thermophilus*, which encodes a lactose permease (*lacS*) and a β-galactosidase (*lacZ*). The *lacSZ* operon, expressed from a library of synthetic promoters, was integrated into the chromosomal attachment site (*attB)* of the *C. glutamicum* strain JS34, a derivative of ATCC13032 (Fig. 1Aa) [33]. For this purpose we used a recently developed integration tool that allows for multiple successive integration events [33]. The outcome was a large number of strains (a library), each expressing the *lacSZ* operon to a different level, thus resulting in different growth rates on lactose. JS46, an isolate with superior growth properties on lactose, was characterized and found to metabolize the glucose moiety of lactose, and accumulate galactose in the medium (Fig. 1Ab). The *galMKTE* operon from *L. lactis* encoding the Leloir pathway, was subsequently introduced into JS46 in the same manner (Fig. 1B), and JS93 was obtained which grew in minimal medium containing lactose without accumulation of galactose.

One advantage of having the lactose genes integrated into the chromosome could be increased genetic stability, the lack of which often is seen when relying on plasmids [32]. To test the stability of JS93, the strain was grown in rich BHI broth containing glucose for approximately 100 generations (using a serial transfer regime), after which the culture was diluted and plated on BHI agar. Sixteen colonies were randomly picked and re-streaked on the BMCG minimal agar, either with lactose or galactose. Additional file 1 shows that all of the colonies retained the ability to grow on both lactose and galactose.

### Enhancing the performance of JS93 through adaptive laboratory evolution

**Fig. 2:**
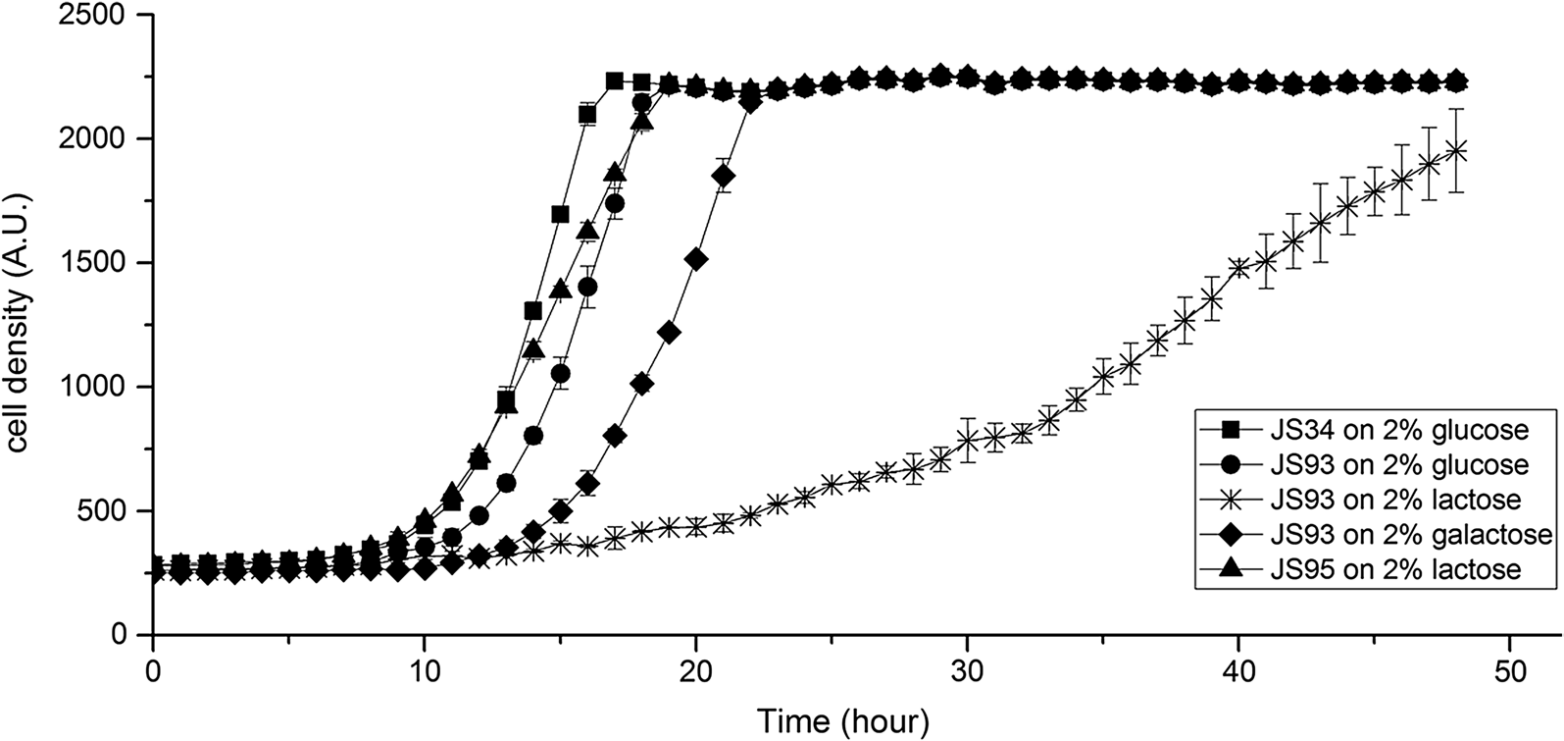
Growth comparisons of JS95, JS93 and JS34 on the BMCG medium with different carbon sources. The experiments were performed using a microbioreactor (Biolector). The standard deviations were calculated from three independent experiments. *AU* arbitrary units, which is the parameter indicating cell density for the Biolector

When galactose was used as the sole carbon source, JS93 displayed fast growth, comparable to that observed on glucose. However, a dramatically slower growth rate was observed on lactose (Fig. 2). The *lacSZ* operon originated from *S. thermophilus*, which could influence the expression and function in *C. glutamicum*. To improve growth on lactose, we decided to apply adaptive laboratory evolution. After a 100-generation evolution on the BMCG medium with lactose, we identified the fast-growing derivative JS95. This strain grew equally fast on lactose, galactose and glucose (Fig. 2).

To reveal the underlying cause of the fast growth, the genome of JS95 was sequenced. Only one single nucleotide variation (SNV) was identified, a C to T substitution located in the integrated *lacS* gene, which resulted in a Pro148 to Leu amino acid change.

### Optimization of a whey-based medium for cultivating *C. glutamicum*

The strain JS95 was able to grow well in minimal medium with lactose. Since the ultimate goal was to create a platform for converting lactose in DWP into value-added chemicals, we decided to test how well JS95 could grow in a DWP-based medium consisting of two times diluted DWP. Even though this medium contained 5% lactose, the cell density of JS95 could only reach a low cell density (OD_600_ = 0.62 ± 0.01) after 24 h of aerobic cultivation (Table 1). We speculated that the DWP medium might be missing some components essential for growth, and decided to test whether adding some of the components found in the BMCG minimal medium (see Additional file 2 for the components of the DWP) could be beneficial. One experiment revealed that by merely adding 2 mg/L MnSO_4_·H_2_O, growth could be significantly improved, and the accumulated biomass increased by a factor of almost 20 (OD_600_ = 16 ± 0.42 (Table 1). By adding 0.02 g/L FeSO_4_·7H_2_O, a marginal improvement was observed, however, no synergistic effect was observed when both Mn^2+^ and Fe^2+^ were added. The single addition of 7 g/L (NH_4_)_2_SO_4_ and trace metals had no effects on promoting the growth on DWP. However, when the medium in addition was supplemented with Mn^2+^ and Fe^2+^, the final cell density attained for JS95 was OD_600_ = 56.80 ± 1.27. The remaining components, e.g. catechol and biotin did not have a stimulatory effect on cell growth (data not shown). The optimal medium was termed DWP-1.

**Table 1:** Growth response of strain JS95 to various supplements on DWP

Addition to DWP^a^	OD_600_ after 24 h aerobic cultivation at 30 °C
None	0.62 ± 0.01
(NH_4_)_2_SO_4_	0.70 ± 0.11
FeSO_4_	3.38 ± 0.43
MnSO_4_	16 ± 0.42
Trace metal mix	0.77 ± 0.04
MnSO_4_, FeSO_4_, trace metal mix	34 ± 1.41
(NH_4_)_2_SO_4_, MnSO_4_, FeSO_4_, trace metal mix	56.8 ± 1.27

The concentration of the nutrients can be found in methods of optimization of whey-based medium for *C. glutamicum* mutant

^a^Dilute the raw DWP according to 1 volume DWP with 1 volume H_2_O

### Ethanol production from delactosed whey permeate using JS122

#### Ethanol production using re-suspended cells in a batch mode

JS95 can efficiently metabolize the lactose in DWP and should be a good platform for valorizing whey waste. To demonstrate this, we decided to modify it further to enable production of ethanol. The pyruvate decarboxylase (PDC) and the alcohol dehydrogenase (ADHB) from *Z. mobilis* were introduced into JS95, and furthermore, *ldhA*, encoding l-lactate dehydrogenase and *ppc*, encoding phosphoenolpyruvate carboxylase, were knocked out to eliminate/reduce lactate and succinate formation respectively, and the resulting strain was designated JS122.

**Fig. 3:**
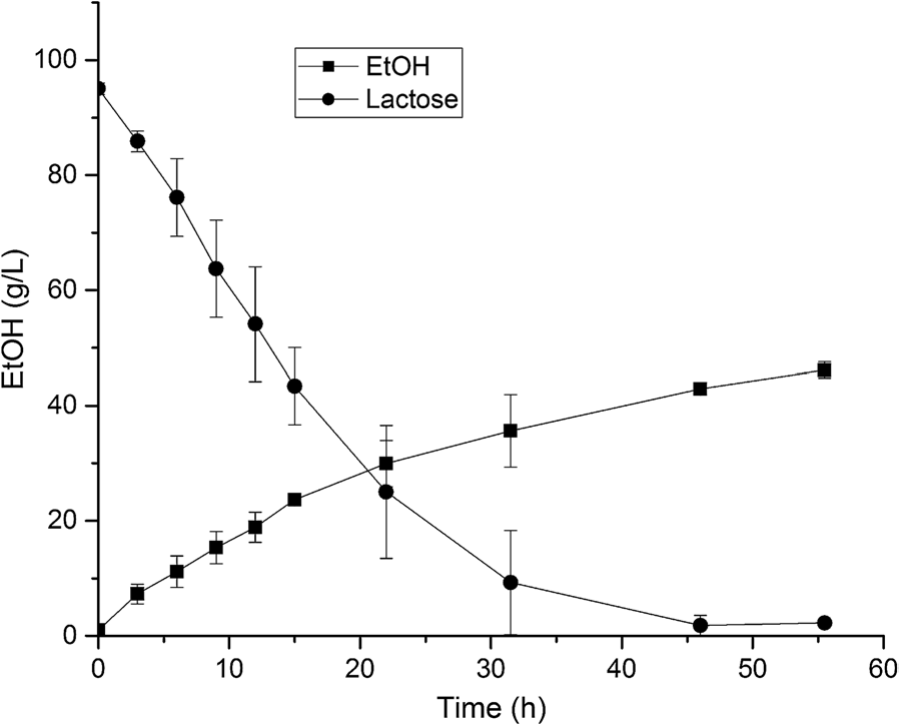
Ethanol batch fermentation by JS122 on DWP under oxygen deprivation conditions. The cell density was 24 g/L CDW. The standard deviations were calculated from three independent experiments

Initially, a two-stage batch fermentation setup was used [29, 30]. For the first stage, the strain JS122 was cultivated aerobically in DWP-1. After stage one, the cells were harvested by centrifugation, resuspended in DWP, after which the fermentation took place under anaerobic conditions. The 100 g/L sugars (95 g/L lactose and 5 g/L galactose) contained in the DWP were completely consumed within 55 h and 46.2 ± 1.4 g/L (5.8 vol.%) ethanol was produced. The yield of ethanol on DWP was 88% of the theoretical yield (Fig. 3). The maximum ethanol production rate was 1.45 g/L/h, which was maintained for the first 15 h (Fig. 3). Glycerol (9.5 ± 1.2 g/L), succinate (1.6 ± 1.0 g/L), and acetate (0.6 ± 0.3 g/L) were found as the major byproducts.

#### Increasing the ethanol titer by lactose feeding

**Fig. 4:**
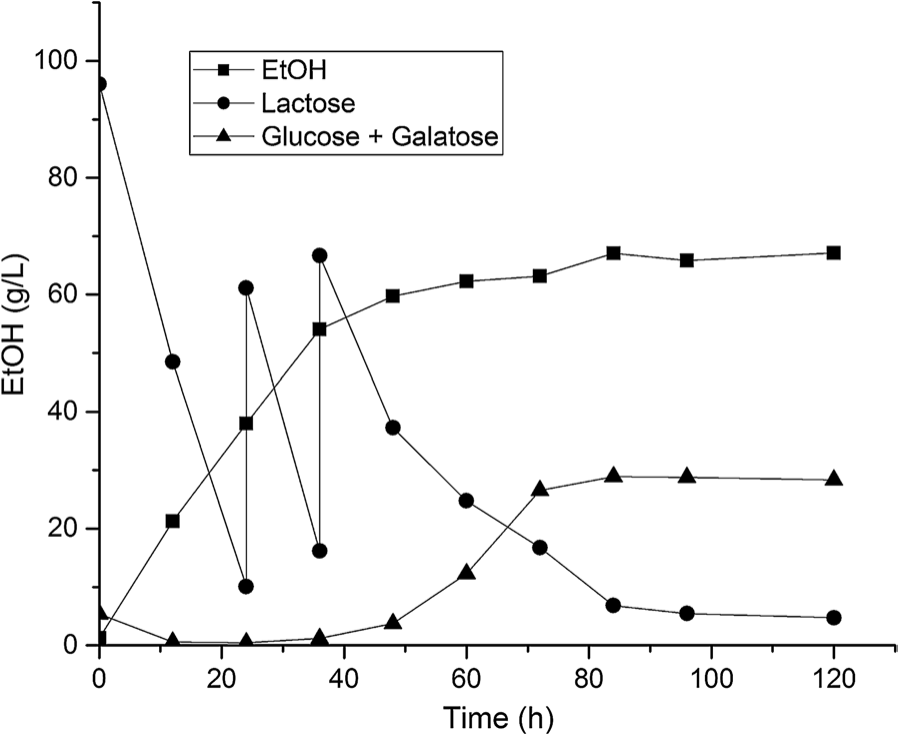
One representative ethanol fed-batch fermentation by JS122 on DWP under oxygen deprivation conditions. DWP contains 95 g/L lactose and 5 g/L galactose, and after 24 h and 48 h, 50 g/L of lactose was added. The cell density was 24 g/L CDW

In order to keep the costs low for the ethanol distillation process, a high ethanol content in the fermentation medium is preferred. Therefore, we investigated whether the ethanol titer could be increased by adding lactose. By using this approach, it was possible to increase the final ethanol titer to 67 g/L, within 120 h (Fig. 4). It was clear that the ethanol production rate decreased dramatically after 48 h, at which point 60 g/L ethanol had accumulated. In addition, secretion of glucose and galactose into the fermentation broth was observed (Fig. 4).

#### Re-use of cells

**Fig. 5:**
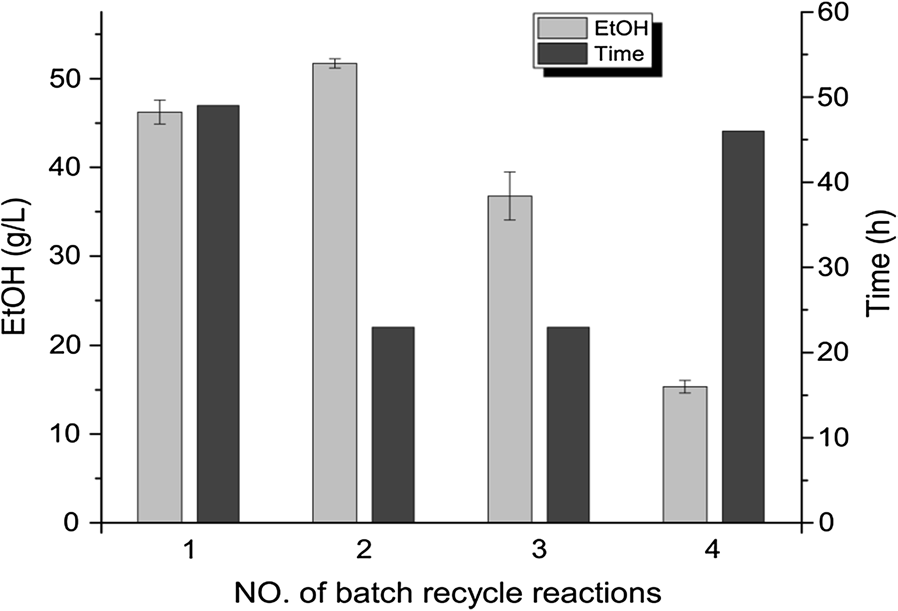
Ethanol production by recycled cells under oxygen deprivation. The left column shows the titer (g/L) of ethanol achieved by each recycle batch reaction. The right column shows the average time (h) of each recycle batch. The standard deviations were calculated from three independent experiments

Potentially, the cells could still be active after the batch fermentation, as the ethanol titer reached was lower than the maximum ethanol tolerance observed in the fed-batch fermentation, and we therefore decided to investigate whether re-use of the cells for additional rounds of ethanol production was possible or not. In the first round, when the ethanol titer reached 46.24 ± 1.35 g/L after 48 h, the cells were centrifuged and re-suspended in the fresh DWP. Ethanol production indeed continued (Fig. 5), and the cells were subsequently used for two additional rounds. Surprisingly ethanol production was faster in the second and third batches, where the titer was 51.72 ± 0.52 g/L and 36.78 ± 2.72 g/L within 23 h respectively (Fig. 5). In the fourth round, production ceased after 46 h, and only 16.71 ± 1.15 g/L ethanol was produced.

## Discussion

For bacteria, there are two prevalent ways in which lactose can be metabolized. For taking up lactose, some bacteria rely on a phosphotransferase transport system (PTS), and the lactose enters the cell as lactose-6-phosphate. Inside the cells, lactose-6-phosphate is hydrolyzed by a 6-phospho-β-galactosidase into glucose and galactose-6-phosphate. Galactose-6P can be further metabolized through the tagatose pathway [34]. In our first attempt to transform *C. glutamicum* into metabolizing lactose, we tried to express the PTS pathway from *L. lactis* in *C. glutamicum*, but were unsuccessful (data not shown). Previously, the lactose permease (LacY/LacS), β-galactosidase (LacZ), and the Leloir pathway (GalMKTE) have been expressed in *C. glutamicum*, thereby enabling growth [35]. However, growth on lactose was poor, and 30% of the cell population lost the ability to metabolize lactose after a 24 h cultivation due to plasmid instability [35]. Under non-selective conditions, e.g., when growing on glucose, this phenomenon was exacerbated, and 75% of the cells could no longer metabolize lactose [35]. It is thus paramount to stabilize the heterologous gene expression in *C. glutamicum*, in order to obtain a stable platform that can be used in large scale. For this work, therefore, we decided to rely on chromosomal integration. We introduced the *lacSZ* and *galMKTE* genes in the strain via site-specific chromosomal integration [33] (Fig. 1). We demonstrated that the strain indeed was stable, by sub-culturing in rich medium containing glucose (Additional file 1). It is possible that the previously observed instability was caused by a metabolic load due to excessive expression of the genes from high-copy number plasmids [36]. In this study we expressed the genes in single-copy from the chromosome, and instead of using strong promoters, we applied synthetic promoters to fine-tune expression [37]. The expression of *lacSZ* and *galMKTE* had no apparent negative effect on fitness, as JS93 grew as fast as the wild-type strain on glucose (Fig. 2).

When the Leloir pathway (GalMKTE) was expressed in the *lacSZ* mutant, fast growth was observed on galactose minimal medium, however, the growth on lactose was slow. The rate-limiting step most likely was either the lactose transport or hydrolysis of lactose by the β-galactosidase [32]. When we exposed the strain to a short-term adaptive evolution on lactose, a fast-growing mutant with a mutation in the lactose permease gene (*lacS*) was obtained. Since no other mutations could be detected, it was clear that the *lacS* mutation was responsible for the fast growth.

It is known that the cell membrane composition can affect the activity of membrane transport proteins, and a good example of this is the lactose permease LacY of *E. coli*, which strictly requires phosphatidyl ethanolamine in order to function [8]. Differences in cell membrane composition between *C. glutamicum* and *S. thermophilus* could possibly explain why JS93 grew slowly on lactose, but further investigation is needed, before any solid conclusions can be reached.

An efficient platform for valorizing DWP should be able to grow well in a DWP based medium without excessive amounts of expensive nutrients added. DWP contains a substantial amount of sugars (lactose and galactose), proteins, vitamins and minerals (Additional file 2) and therefore we first attempted to cultivate JS95 in pure DWP, however, only little growth was possible. The main nitrogen source in whey permeate is casein and whey proteins, and a low concentration of free amino acids [38]. It seemed plausible that access to nitrogen sources limited growth on DWP, since *C. glutamicum* lacks a protease to digest the milk proteins. However, supplementation with (NH_4_)_2_SO_4_ could not stimulate growth, which indicates that other factors were limiting. After testing various components present in the BMCG minimal medium, which supports efficient growth of *C. glutamicum* to high cell densities, it was found that the addition of Mn^2+^ had a dramatic effect (Table 1). For obligate aerobes such as *C. glutamicum*, Mn^2+^ is an important divalent cation for many enzymes involved in various aerobic processes. One well-characterized enzyme is superoxide dismutase, which is essential for protecting the cell against reactive oxygen species (ROS). The activity of superoxide dismutase in *C. glutamicum* is strictly dependent on manganese, and the replacement with other cations results in inactivation [39]. Beside Mn^2+^, Fe^2+^ is another important cation involved in cellular metabolism and growth [40, 41]. We also observed that Fe^2+^ alone had a minor stimulatory effect on biomass formation. The major cations present in milk are Ca^2+^ (19 mg/L), Mg^2+^ (2 mg/L), K^+^ (29 mg/L) and Na^+^ (10 mg/L) [42]. The low concentrations of Mn^2+^ (0.022 mg/L) and Fe^2+^ (0.45 mg/L) in milk, also in DWP, are apparently not sufficiently high to allow for efficient growth of *C. glutamicum*. Perhaps the concentrations of these metal ions are somehow further reduced in the processing steps leading to DWP, which seems likely as Mn^2+^, Fe^2+^ and other trace minerals have been reported to be associated with the milk proteins [43]. Both trace minerals and NH_4_^+^ were found not to be critical for growth on DWP, but the addition was important to attain a high cell density (Table 1). This was probably due to the presence of a moderate amount of peptides, amino acids, and trace metals in whey permeate (Additional file 2).

To achieve ethanol production from the lactose in DWP, we deleted the *ldh* and *ppc* genes to eliminate or reduce byproduct formation, and introduced the *pdc* and *adhB* genes from *Z. mobilis*, to channel the metabolic flux towards ethanol. Using resuspended cells under anaerobic conditions resulted in a high-titer (46 g/L) and high-yield (0.88 of the maximum theoretical yield) ethanol production on DWP. In previous studies, the main by-product reported for *C. glutamcim* strains able to produce ethanol was succinic acid, which necessitated pH control during fermentation [29, 30]. In our study, we did not observe a drop in pH, and the dominating byproduct was glycerol. Both the formation of succinic acid and glycerol results in the consumption of one net NADH when glucose is metabolized via glycolysis [44]. Production of ethanol by *C. glutamicum* from DWP is thus a more simple process as there is no need for pH control.

It is well-known that ethanol, at high concentrations, can permeabilize bacterial membranes [45]. Ethanol eventually will block cell metabolism, e.g., glycolysis, and thus have an effect on non-growing cells as well [45]. We managed to reach 60 g/L ethanol after 60 h, when using the lactose feeding approach. After 48 h glucose and galactose started to accumulate in the fermentation broth, which probably was an effect of permeabilization and blocking of glycolysis (Fig. 4). Jojima et al. surprisingly reported 119 g/L ethanol from glucose, using *C. glutamicum* as well, however, they used 2.5 times more cells (60 g CDW/L cells) for the production, which is a drawback costwise. It appears that the biomass concentration is of key importance for final ethanol titer.

We also tried to reuse the cells in order to reduce costs. The cells could be used for three batches of ethanol production without loss of activity (Fig. 5). Ethanol production in the first batch was significantly slower compared to the production in the second and third batch, which could be due to the transition from aerobic growth to anaerobic fermentation where glycolytic genes need to be induced [46]. The final ethanol titer reached in the first three cell recycle fermentations was close to the maximum ethanol tolerance indicated by the fed-batch fermentation. Exposure to a high concentration of ethanol during the three cycles is the most likely reason for why the cells gradually became inactive and started to hydrolyze lactose (Fig. 5). The ethanol productivity obtained in the first three cell recycle experiments were 0.96 g/L/h, 2.2 g/L/h, and 1.6 g/L/h, respectively. These productivities are comparable with those found in other studies. Using a recombinant *S. cerevisiae* flocculent strain, Guimarães et al. achieved ethanol productivities of 1.5–2 g/L/h with a yield of 78–84% of the theoretical in shake flask fermentations, which was higher than in the bioreactor fermentations (< 60%) [47]. The ethanol productivity of an engineered *L. lactis* growing on whey permeate medium supplemented with yeast extract was 0.96 g/L/h [26]. Inui et al. demonstrated that ethanol productivity, as expected, increased in proportion to cell density for *C. glutamicum* [29]. An alternative to increasing cell density could be to increase glycolytic flux, which also has been reported to be feasible for *C. glutamicum* [30,48].

## Conclusion

In conclusion, we have presented a novel process for valorizing dairy waste. The process relies on an engineered microbial platform based on *C. glutamicum*, and a low cost modification of the dairy waste feedstock to allow for efficient amplification of the microbial platform. For engineering the platform we relied on a recently developed site-specific chromosomal integration tool for integrating lactose- and galactose-utilization pathways into the chromosome for stabilization purposes. Adaptive Laboratory Evolution and optimization of the DWP-based medium was carried out to facilitate fast and efficient biomass accumulation. We have demonstrated the potential of this platform by producing ethanol from DWP in a time and cost efficient manner. To our knowledge, this is the first report demonstrating efficient ethanol production from the lactose contained in dairy waste using an engineered *C. glutamicum* strain. We have not only constructed a novel platform, rather we have developed a process that efficiently can transform an existing dairy waste stream into ethanol and potentially a plethora of other useful compounds.

## Materials and methods

### Bacterial strains, plasmids

All the bacterial strains and plasmids used in this study are listed in Table 2.

**Table 2:** Strains and plasmids

Strain/plasmid	Description/function	References
Strains		
*C. glutamicum* ATCC13032	Wild-type	ATCC
JS34	*C. glutamicum* inserted with *att*B site on the chromosome	[33]
JS45-A, B, C, D, E	JS34 site-specific integrated the *lacSZ* operon, KanR	This study
JS46	Marker excised via *lox*66–*lox*71 recombination from JS45-E	This study
JS86-A, B, C, D	JC114-E site-specific integrated the *galMKTE* operon, KanR	This study
JS93	Marker excised via *lox*66–*lox*71 recombination from JS86-D	This study
JS95	Evolved JS93	This study
JS99	JS95Δ*ldh*	This study
JS112	JS99Δ*ppc*	This study
JS122	JS112 bearing pJS115, SpecR	This study
*S. thermophilus*	Source of the *lacSZ* operon	[35]
*L. lactis* MG 1363	Source of the *galMKTE* operon	[53]
*E. coli* TOP 10	Transformation host	Lab stock
Plasmids		
pK18mobsacB	Used for deleting *ldh* and *ppc* genes, KanR	[50]
pJS31	Used for site-specific integration, KanR	[33]
pAL347	*E. coli*–*C. glutamicum* expression shutter vector, SpecR	[57]
pJS115	A derivative from pAL347 for ethanol producing, SpecR	This study

### Growth medium and conditions

*Escherichia coli* strains were grown aerobically in Luria–Bertani broth (LB) [49] at 37 °C, and *C. glutamicum* strains were cultivated in Brain Heart Infusion broth (BHI) at 30 °C with 200-rpm shaking [50]. When appropriate, kanamycin was added to a concentration of 50 μg/mL for *E. coli* and 25 μg/mL for *C. glutamicum,* and spectinomycin was used at a concentration of 100 μg/mL for *E. coli* and 50 μg/mL for *C. glutamicum*. Cell growth was monitored by measuring the optical density at 600 nm (OD_600_) of the culture broth using a UV1800 spectrophotometer (Shimadzu, Japan).

### Construction of the lactose- and galactose-utilizing *C. glutamicum* strains

The *C. glutamicum* derivatives with the chromosomally integrated lactose and galactose metabolic pathway genes were constructed via two successive site specific integrations as described previously [33]. For constructing a *C. glutamicum* strain that can use the glucose moiety of lactose, the *lacSZ* operon, encoding the lactose transporter and β-galactosidase from *Streptococcus thermophilus* (*S. thermophilus*) [35, 51], was PCR amplified using the primers p-lacSZ-F and p-lacSZ-R (Additional file 3). The primer p-lacSZ-F contained a degenerate synthetic promoter sequence functioning in *C. glutamicum* [37]. The resulting PCR product was digested with the restriction enzymes *Bam*HI and *Xho*I, and then cloned into the vector pJS31 treated with the same enzymes. The expression library pJS31-*lacSZ* was first propagated in *E. coli*, and then transformed into the *C. glutamicum* strain JS34 with the expression cassette of the TP901-1 integrase as previously described [33]. Formed colonies with large size, indicating good growth on the BMCG-lactose agar plate, were chosen for further growth assessment on the BMCG medium [52] containing 1% lactose. JS45-E that grew fast on lactose was selected for marker excision using the Cre recombinase as previously described [33]. The marker-free strain was designated as JS46. The galactose metabolic pathway genes were introduced into the chromosome of *C. glutamicum* strain JS46 in a similar manner. The *L. lactis* MG1363 Leloir pathway operon [53] was amplified using the primers p-*galMKTE*-F and p-*galMKTE*-R, and then cloned to the vector pJS31. The aforementioned procedure for gene integration and marker excision was carried out. The resulting marker-free strain, JS93, could use lactose or galactose as sole carbon source.

### Procedure for adaptive laboratory evolution

The evolution was conducted using a serial-transfer regime with the strain JS93, according to the procedure previously described [54]. Briefly, a single colony of JS93 was inoculated into a test tube containing 5 mL BMCG medium with 2% lactose and cultivated at 30 °C with 200-rpm shaking. When the culture entered the stationary phase, 0.05 mL culture was transferred into a new test tube with the same medium, which is equal to 6.64 generations of growth. Each week, a copy of the culture was stored in 25% glycerol at − 80 °C. The performance of the culture on lactose was regularly checked. After a 100-generation adaptive evolution, culture from the final tube was streaked on the BMCG lactose plate, and one fast-growing mutant was isolated which was designated JS95.

### Genome sequencing

Genomic DNA of the mutant was purified using DNeasy Blood & Tissue Kit (Qiagen) and the quality was checked by DNA electrophoresis and NanoDrop 1000 (Thermo Fisher Scientific) analysis. Genome sequencing was performed by Beijing Genomics Institute (BGI) according to the protocol previously described [55]. CLC Genomics Workbench (Qiagen) was used for mapping the reads, SNP (single nucleotide polymorphism), DIP (deletion–insertion polymorphism) detection, and identification of genomic rearrangement using the published genome sequence of *C. glutamicum* ATCC 13032 as the Ref. [56].

### Optimization of whey-based medium for *C. glutamicum* mutant

For testing the effect of different additions, (NH_4_)_2_SO_4_ (7 g/L), FeSO_4_·7H_2_O (20 mg/L), MnSO_4_·H_2_O (2 mg/L) and the trace element mix (0.4 µM H_3_BO_3_, 0.003 µM (NH_4_)_6_Mo_7_O_24_, 0.01 µM ZnSO_4_, 0.01 µM CuSO_4_, 0.08 µM MnCl_2_, 0.03 µM CoCl_2_) were added into the two-times diluted DWP. The *C. glutamicum* strain JS95 was first aerobically cultivated at 30 °C in 5 mL BHI medium for 12-h (200-rpm) and then 8 μL preculture was inoculated into 800 μL the whey-based medium. The growth experiment was performed on Biolector (M2p-labs, Germany) with 1500-rpm shaking. After 24-h aerobic cultivation, the optical densities (OD_600_) were measured on a UV1800 spectrophotometer (Shimadzu, Japan).

### Construction of ethanol-producing strain

For JS95, the deletion of *ldhA* and *ppc* were conducted via a two-step homologous recombination procedure as described previously using the vector pK18mobsacB [50]. A codon-optimized DNA fragment containing the *pdc* and the *adhB* gene, expressed from the *C. glutamicum ldhA* promoter, was synthesized by GenScript (Additional file 4), and then cloned into pAL347 [57] to generate pJS115. pJS115 was transformed into *ΔldhA*–Δ*ppc* mutant of JS95 by electroporation, and the resulting strain was designated as JS122.

### Conditions for ethanol production

For ethanol production, *C. glutamicum* JS122 was aerobically cultivated at 30 °C for 12–16-h in a 1-L flask containing 200 mL of the two times diluted DWP supplemented with 7 g/L (NH_4_)_2_SO_4_, 20 mg/L FeSO_4_·7H_2_O, and 2 mg/L MnSO_4_·H_2_O and the trace element mix. Cells were harvested by centrifugation (5000×*g*, 10 min), and resuspended in DWP to a cell density of 24 g CDW/L. The cell suspension was subsequently incubated under anaerobic conditions with 100-rpm magnetic stirring to keep the cell suspension homogeneous. For reuse of cells, the suspension was centrifuged (5000×*g*, 10 min), and the pellet was resuspended in fresh DWP.

### Analytical techniques

HPLC analysis of the fermentation broth was carried out using an Ultimate 3000 high-pressure liquid chromatography system (Dionex, Sunnyvale, USA) equipped with an Aminex HPX-87H column (Bio-Rad, Hercules, USA) and a Shodex RI-101 detector (Showa Denko K.K., Tokyo, Japan). The column oven temperature was set to 60 °C. H_2_SO_4_ (5 mM) was used as the mobile phase, and the flow rate was 0.5 mL/min.

## Additional files

**Additional file 1.** The genetic stability of JS93.

**Additional file 2.** The components of delactose whey permeate.

**Additional file 3.** Primers used in this study.

**Additional file 4.** The sequences of the codon-optimized DNA fragment containing the *pdc*, *adhB* genes, and the *C. glutamicum ldhA* promoter.

## Abbreviations

DWP: delactose whey permeate
LB: Luria–Bertani broth
BHI: brain heart infusion broth
*lacSZ*: genes encoding lactose permease and β-galactosidase
*galMKTE*: leloir pathway operon
*pdc*: gene encoding pyruvate decarboxylase
*adhB*: gene encoding alcohol dehydrogenase
*ldhA*: gene encoding lactate dehydrogenase
*ppc*: gene encoding phosphoenolpyruvate carboxylase
OD_600_: optical density at wavelength of 600 nm
CDW: cell dry weight
