# Supplementary figures and images for: Development of a novel, robust and cost-efficient process for valorizing dairy waste exemplified by ethanol production
Source: Microb Cell Fact. 2019 Mar 11;18:51. doi: 10.1186/s12934-019-1091-3 (PMC6410493; doi:10.1186/s12934-019-1091-3)

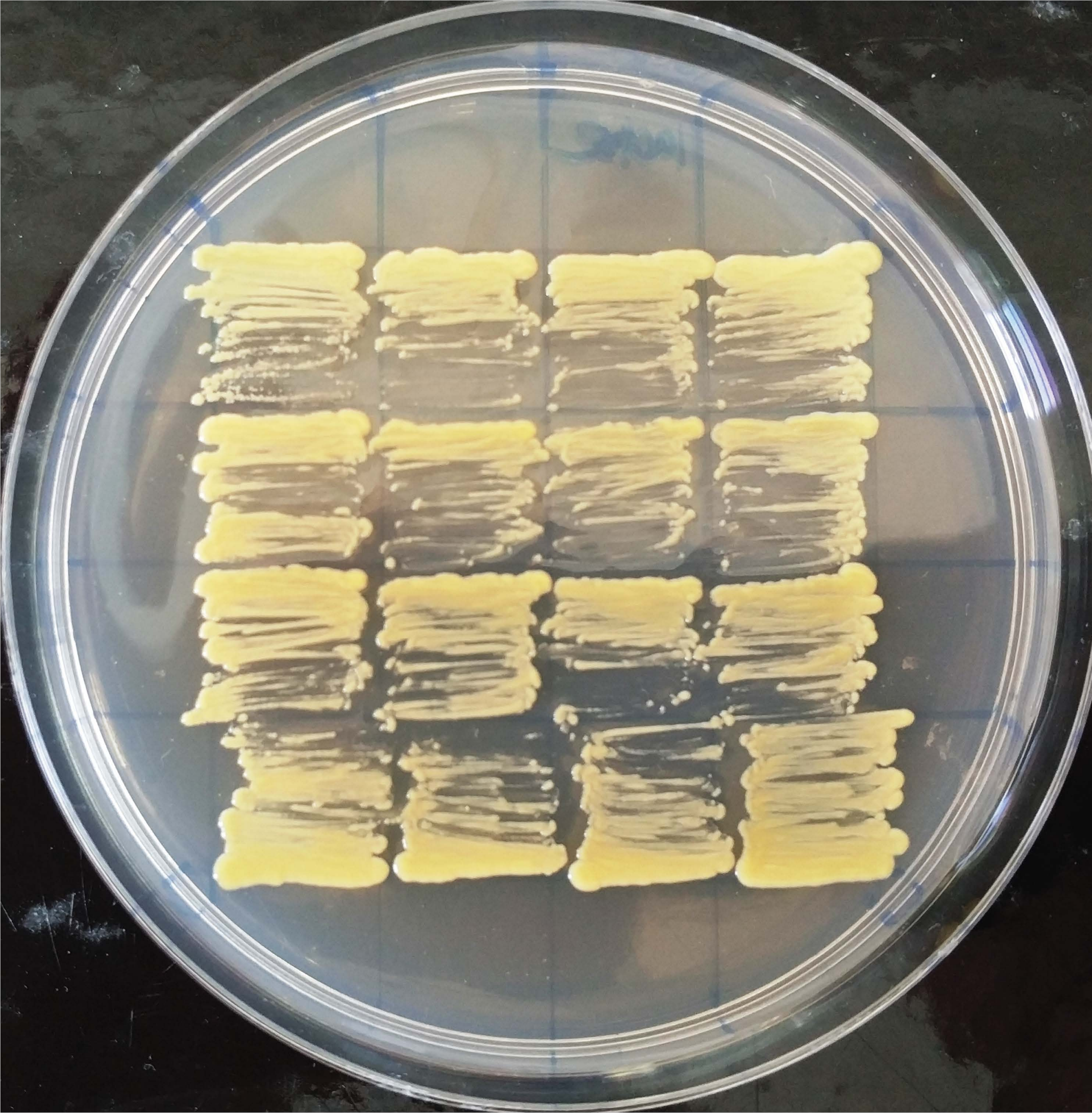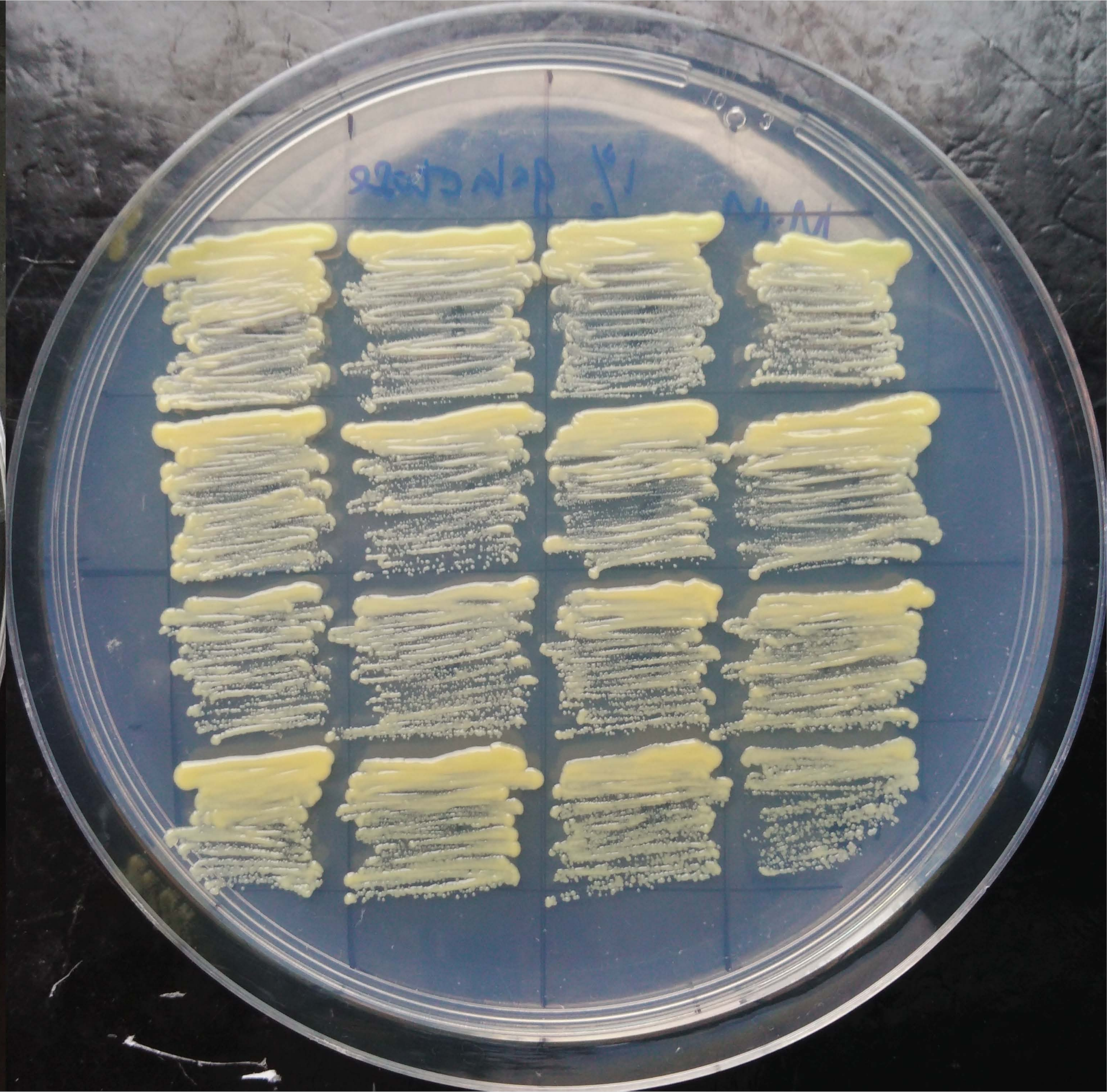

Supplement: Supplementary file 1 — Additional file 1. The genetic stability of JS93. [file 12934_2019_1091_MOESM1_ESM.pdf]
