# Supplementary material for: Development of a novel, robust and cost-efficient process for valorizing dairy waste exemplified by ethanol production
Source: Microb Cell Fact. 2019 Mar 11;18:51. doi: 10.1186/s12934-019-1091-3 (PMC6410493; doi:10.1186/s12934-019-1091-3)
Supplement: Supplementary file 3 — Additional file 3. Primers used in this study. [file 12934_2019_1091_MOESM3_ESM.docx]

| Name | Sequences |
| --- | --- |
| *ldh*-up-F | CCCC**AAGCTT**CTCTCAATTAGGAGCTTGACCTC |
| *ldh*-up-R | ACGC**GTCGAC**TTTCGATCCCACTTCCTGAT |
| *ldh*-down-F | ACGC**GTCGAC**ATCTTTGGCGCCTAGTTGGC |
| *ldh*-down-R | CCCG**GAATTC**GGTTTCGTCGCGGGTTACTC |
| *ppc*-up-F | ACGACGGCCAGTGCCAAGCTTGCATGCCTGCAGGTCGACTAGGGGTCCATGTTAAAGG |
| *ppc*-up-R | ATGACTGATTTTTTACGCGAACCATGAACGGTCTTTCC |
| *ppc*-down-F | GGAAAGACCGTTCATGGTTCGCGTAAAAAATCAGTCAT |
| *ppc*-down-R | ATGATTACGAATTCGAGCTCGGTACCCGGGGATCCTCTAGACAAGCTTGAGGTCACTTTC |
| *lacSZ*-F | ACGC**GGATCC**TTGACANNNNNNNNNNNNTGNGNTAYAATGGNNNAGTGCATTCAAATAATAGGAGGTTTC |
| *lacSZ*-R | ACCG**CTCGAG**CTAATTTAGTGGTTCAATCA |
| *glMKTE*-F | TGCAGGACAGTCGACTTGACCCGGGAAAAGCATGCTACTTTTGACANNNNNNNNNNNNTGNGNTAYAATGGNNNAGTGCAAGTTGACCTCAGGTTAGCC |
| *glMKTE*-R | TCAATTGACCAAGTACTTCTTCTCGAGTTTGATTCTCTAGTCAGTAGCCTTTTGGATGAC |

Table S1 Primers used in this study.
