# Supplementary material for: Development of a novel, robust and cost-efficient process for valorizing dairy waste exemplified by ethanol production
Source: Microb Cell Fact. 2019 Mar 11;18:51. doi: 10.1186/s12934-019-1091-3 (PMC6410493; doi:10.1186/s12934-019-1091-3)
Supplement: Supplementary file 4 — Additional file 4. The sequences of the codon-optimized DNA fragment containing the pdc, adhB genes, and the C. glutamicum ldhA promoter. [file 12934_2019_1091_MOESM4_ESM.pdf]

pdc adhB sequences

TTTAAGAAAGGTGTGTTTCACACATGTCCTATACCGTTGGCACCTATCTGGCGGAGCGTCTCGTTCAAATCGGTCTCAA
GCACCACTTCGCAGTTGCAGGCGACTACAATCTCGTGCTGCTCGACAACCTGCTCCTGAACAAGAACATGGAACAGGT
CTACTGCTGCAACGAGCTGAACTGCGGCTTCTCCGCAGAAGGTTACGCACGTGCAAAGGGTGCAGCAGCAGCAGTGG
TCACCTACTCCGTGGGCGCTCTCTCCGCATTCGATGCCATCGGCGGCGCTTACGCAGAGAACCTCCCAGTGATCCTGAT
CTCCGGCGCTCCAAACAACAACGATCACGCCGCTGGCCACGTCCTCCACCACGCACTGGGCAAGACCGACTACCACTA
CCAGCTGGAAATGGCAAAGAACATCACCGCAGCCGCTGAGGCCATCTACACCCCAGAAGAGGCCCCAGCTAAGATCG
ACCACGTGATCAAGACCGCCCTCCGCGAAAAGAAGCCAGTCTACCTGGAGATCGCATGCAACATCGCATCCATGCCAT
GCGCAGCACCAGGTCCAGCATCCGCCCTGTTCAACGATGAAGCTTCCGACGAGGCATCCCTCAACGCAGCAGTGGAA
GAAACCCTGAAGTTCATCGCTAACCGCGACAAGGTCGCAGTGCTGGTGGGTTCCAAGTTGCGTGCCGCTGGCGCAGA
AGAGGCAGCCGTGAAGTTCGCTGATGCACTGGGCGGCGCCGTGGCTACCATGGCAGCAGCAAAGTCCTTCTTCCCAG
AAGAGAACCCACACTACATCGGCACCTCCTGGGGCGAAGTGTCCTACCCAGGCGTCGAAAAGACCATGAAGGAAGCA
GATGCCGTGATCGCTCTGGCACCAGTCTTCAACGACTACTCCACCACCGGCTGGACCGATATCCCAGACCCAAAGAAG
CTCGTGCTGGCCGAACCACGCTCCGTGGTCGTGAACGGCATCCGCTTCCCATCCGTGCACCTCAAGGATTACCTCACCC
GCCTGGCTCAGAAGGTGTCCAAGAAGACCGGCGCACTGGACTTCTTCAAGTCCCTCAACGCCGGCGAACTGAAGAAG
GCTGCACCAGCCGATCCATCCGCTCCACTCGTGAACGCTGAAATCGCACGTCAGGTGGAGGCACTCCTGACCCCAAAC
ACCACCGTGATCGCTGAAACCGGCGACTCCTGGTTCAACGCACAGCGCATGAAGCTGCCAAACGGCGCCCGCGTCGA
ATACGAGATGCAGTGGGGTCACATCGGTTGGTCCGTTCCAGCCGCTTTCGGTTACGCAGTGGGTGCTCCAGAACGTC
GCAACATCCTCATGGTGGGCGATGGCTCCTTCCAGCTGACCGCACAGGAAGTGGCCCAGATGGTCCGCCTCAAGCTG
CCAGTGATCATCTTCCTCATCAACAACTACGGCTACACCATCGAAGTCATGATCCACGATGGCCCATACAACAACATCA
AGAACTGGGACTACGCAGGCCTGATGGAGGTGTTCAACGGTAACGGTGGCTACGATTCCGGTGCAGGCAAGGGCCT
CAAGGCTAAGACCGGCGGCGAACTGGCTGAGGCTATCAAGGTGGCACTCGCCAACACCGATGGCCCAACCCTGATCG
AATGCTTCATCGGCCGCGAGGACTGCACCGAAGAGCTGGTCAAATGGGGCAAGCGTGTCGCCGCAGCCAACTCTCGT
AAGCCAGTCAATAAGCTCCTGTAAAAACTTTTTAAGAAAGGTGTGTTTCACACATGGCCAGCAGCACCTTTTACATCC
CCTTTGTGAACGAAATGGGCGAAGGCAGCCTGGAAAAAGCCATCAAAGACCTGAACGGCAGCGGCTTTAAAAACGCC
CTGATCGTGAGCGATGCCTTTATGAACAAAAGCGGCGTGGTGAAACAGGTGGCCGATCTGCTGAAAGCCCAGGGCAT
CAACAGCGCCGTGTACGATGGCGTGATGCCCAACCCCACCGTGACCGCCGTGCTGGAAGGCCTGAAAATCCTGAAAG
ATAACAACAGCGATTTTGTGATCAGCCTGGGCGGCGGCAGCCCCCACGATTGCGCCAAAGCCATCGCCCTGGTGGCC
ACCAACGGCGGCGAAGTGAAAGATTACGAAGGCATCGATAAAAGCAAAAAACCCGCCCTGCCCCTGATGAGCATCAA
CACCACCGCCGGCACCGCCAGCGAAATGACCCGCTTTTGCATCATCACCGATGAAGTGCGCCACGTGAAAATGGCCAT
CGTGGATCGCCACGTGACCCCCATGGTGAGCGTGAACGATCCCCTGCTGATGGTGGGCATGCCCAAAGGCCTGACCG
CCGCCACCGGCATGGATGCCCTGACCCACGCCTTTGAAGCCTACAGCAGCACCGCCGCCACCCCCATCACCGATGCCT
GCGCCCTGAAAGCCGCCAGCATGATCGCCAAAAACCTGAAAACCGCCTGCGATAACGGCAAAGATATGCCCGCCCGC
GAAGCCATGGCCTACGCCCAGTTTCTGGCCGGCATGGCCTTTAACAACGCCAGCCTGGGCTACGTGCACGCCATGGCC
CACCAGCTGGGCGGCTACTACAACCTGCCCCACGGCGTGTGCAACGCCGTGCTGCTGCCCCACGTGCTGGCCTACAAC
GCCAGCGTGGTGGCCGGCCGCCTGAAAGATGTGGGCGTGGCCATGGGCCTGGATATCGCCAACCTGGGCGATAAAG
AAGGCGCCGAAGCCACCATCCAGGCCGTGCGCGATCTGGCCGCCAGCATCGGCATCCCCGCCAACCTGACCGAACTG
GGCGCCAAGAAGGAAGATGTGCCCCTGCTGGCCGATCACGCCCTGAAAGATGCCTGCGCCCTGACCAACCCCCGCCA
GGGCGATCAGAAAGAAGTGGAAGAACTGTTTCTGAGCGCCTTTTAG
